# Galectin-1 levels do not predict outcomes in undifferentiated arthritis: a two-year prospective observational study

**DOI:** 10.3389/fimmu.2026.1757168

**Published:** 2026-02-11

**Authors:** Cristina Valero-Martínez, Marisa Pardines-Ortiz, Nuria Montes, Ana M. Ortiz, Rosario García-Vicuña, Isidoro González-Alvaro, Ana Triguero-Martínez

**Affiliations:** 1 Rheumatology Department, Hospital Universitario La Princesa, Instituto de Investigación Sanitaria La Princesa (IIS-IP), Madrid, Spain; 2 Medicine Department, School of Medicine, Universidad Autónoma of Madrid, Madrid, Spain; 3 Immunometabolism and Inflammation Laboratory, Hospital General Universitario Gregorio Marañón, Instituto de Investigación Sanitaria Gregorio Marañón, Madrid, Spain

**Keywords:** biomarkers, connective tissue disease, early arthritis, galectin-1, rheumatoid arthritis, spondyloarthritis, undifferentiated arthritis

## Abstract

**Introduction:**

Undifferentiated arthritis (UA) represents an early and heterogeneous phase of inflammatory joint disease that may evolve into defined rheumatic conditions. This phase is characterized by immune dysregulation with loss of tolerance and early autoantibody production. Despite extensive research, reliable biomarkers for early diagnosis and prognosis remain lacking. Galectin-1 (Gal-1), an immunomodulatory lectin with anti-inflammatory properties, has been reported to be elevated in rheumatoid arthritis (RA) and systemic lupus erythematosus (SLE). This study aimed to evaluate the diagnostic and prognostic value of serum Gal-1 levels in UA patients over a two-year follow-up.

**Methods:**

This study analyzed patients from the observational PEARL study, that were classified as UA at baseline visit. After four standardized visits (0, 6, 12 and 24 months), in which biological samples, clinical, laboratory, and treatment data were systematically collected, a definitive diagnosis was established. Serum Gal-1 levels were measured by ELISA at all visits and analyzed in relation to clinical characteristics, final diagnosis, disease activity, and treatment intensity. Multivariate regression models were adjusted for relevant confounders.

**Results:**

A total of 139 patients were included. After two years, 44.6% (n=62/139) progressed to a chronic inflammatory rheumatic disease, mostly RA (67%; n=42/62), while 40.2% (n=56/139) remained as persistent UA. Baseline Gal-1 levels were not associated with seropositivity, functional disability or treatment intensity. A non-significant trend toward lower Gal-1 levels was observed in patients evolving to spondyloarthritis (SpA) whereas no major differences were observed among other diagnostic groups. Over the two-year follow-up, Gal-1 levels remained largely stable across all diseases and did not correlate with disease activity despite significant clinical improvement.

**Conclusion:**

Overall, Gal-1 showed stable serum levels irrespective of disease activity or outcome, with limited predictive or monitoring value in UA. Lower concentrations observed in SpA suggest disease-specific modulation.

## 1 Introduction

The current understanding of undifferentiated arthritis (UA) suggests that it represents an early, heterogeneous phase of inflammatory joint disease influenced by genetic susceptibility and environmental triggers, leading to immune dysregulation and loss of tolerance (1). This phase is often marked by the emergence of autoantibodies and can progress to various defined rheumatic conditions, such as rheumatoid arthritis (RA), spondyloarthritis (SpA) or connective tissue diseases (CTD), or may resolve spontaneously (1–3). UA is typically defined as inflammatory arthritis that does not fulfill classification criteria for specific rheumatic diseases, reflecting significant clinical variability and uncertainty in long-term outcomes (2, 3).

In recent years, there has been growing interest in identifying diagnostic and prognostic biomarkers capable of predicting, at early stages, which patients presenting with inflammatory musculoskeletal symptoms (with or without autoantibodies) will progress to established rheumatic diseases. Several serum and tissue biomarkers have been explored in this setting, particularly in early RA. Acute-phase reactants such as C-reactive protein (CRP) and serum amyloid A (SAA), synovial and cartilage-derived molecules including matrix metalloproteinases—most notably MMP-3—and inflammatory proteins such as calprotectin have been associated with synovial inflammation, disease activity, and structural progression in early inflammatory arthritis (4, 5). In addition, a range of autoantibodies beyond rheumatoid factor (RF) and anti–citrullinated protein antibodies (ACPA), including anti-carbamylated protein (anti-CarP) and anti-PAD4 antibodies, as well as cartilage degradation products such as COMP and CTX-II, have shown potential value in predicting progression from UA to RA and long-term radiographic damage (4–6). However, despite encouraging findings in selected cohorts, most of these biomarkers have demonstrated limited incremental value over established clinical, serological, and imaging tools, with inconsistent performance across independent populations and disease stages, supporting the ongoing need to identify novel biomarkers for early disease stratification.

Galectins represent a conserved family of β-galactoside-binding proteins, characterized by their specific recognition of poly-N-acetyllactosamine glycoconjugates through evolutionarily conserved carbohydrate recognition domains (CRDs) (7). This family includes at least 15 mammalian members, each containing one or two ~130-amino-acid CRDs, and exhibits dual immunomodulatory capabilities. By cross-linking glycoconjugates, individual galectins can exert either pro-inflammatory or anti-inflammatory effects, thereby modulating various stages of both acute and chronic inflammatory responses. This functional diversity arises from their broad cellular distribution and their ability to interact with distinct molecular partners within immune signaling pathways (7, 8).

Galectin-1 (Gal-1), a 14 kDa galectin capable of forming homodimers, is prominently expressed in key immune cell populations, including inflammatory macrophages, activated T cells, regulatory T cells, and tolerogenic dendritic cells. Its potent anti-inflammatory effects emerge through the coordinated suppression of both arms of the immune response, which have been extensively characterized in experimental models (9, 10). Thus, it can induce apoptosis in activated lymphocytes (particularly Th1 and Th17 cells), promote Th2 polarization, and inhibit the production of inflammatory mediators such as IL-2, IFN-γ, and TNF-α. Gal-1 has been proposed as a potential diagnostic biomarker for various inflammatory autoimmune diseases, including systemic lupus erythematosus (SLE), RA, Sjögren’s syndrome or systemic sclerosis (11). In the context of RA, elevated circulating levels of Gal-1 have been reported compared to healthy controls (11, 12). This increase may reflect an endogenous attempt to counterbalance ongoing inflammation, given Gal-1’s known immunosuppressive properties (8–10). However, despite its promising profile, further studies are needed to validate its clinical utility. In this regard, this work aims to determine if Gal-1 serum levels can be proposed as a biomarker for early diagnosis in patients with pre-RA/UA, or for monitoring disease activity and treatment response.

## 2 Methods

### 2.1 Design, patients and samples

This is a secondary analysis of the Princesa Early Arthritis Register Longitudinal (PEARL) study, an ongoing prospective study at Hospital Universitario La Princesa (Madrid, Spain). PEARL study enrols consecutive patients referred to the Early Arthritis Clinic for specialist evaluation of new-onset inflammatory arthritis, defined by the presence of at least one clinically swollen joint, evaluated by a rheumatologist, with symptom duration of less than one year.

Patients included in PEARL undergo a standardized and protocolized follow-up with predefined visits at baseline, 6, 12, 24 months and 5 years, during which socio-demographic, clinical, laboratory, and therapeutic data are systematically collected and recorded in an electronic database. To ensure consistency of clinical assessment, the protocolized study visits are performed by the same two rheumatologists (AMO and IG-A). Biological samples are obtained at each visit and stored in the IIS-Princesa Biobank (ISCIII B.0000763). All samples are processed following standard operating procedures with the appropriate approval of the Ethics and Scientific Committees.

**Figure 1:**
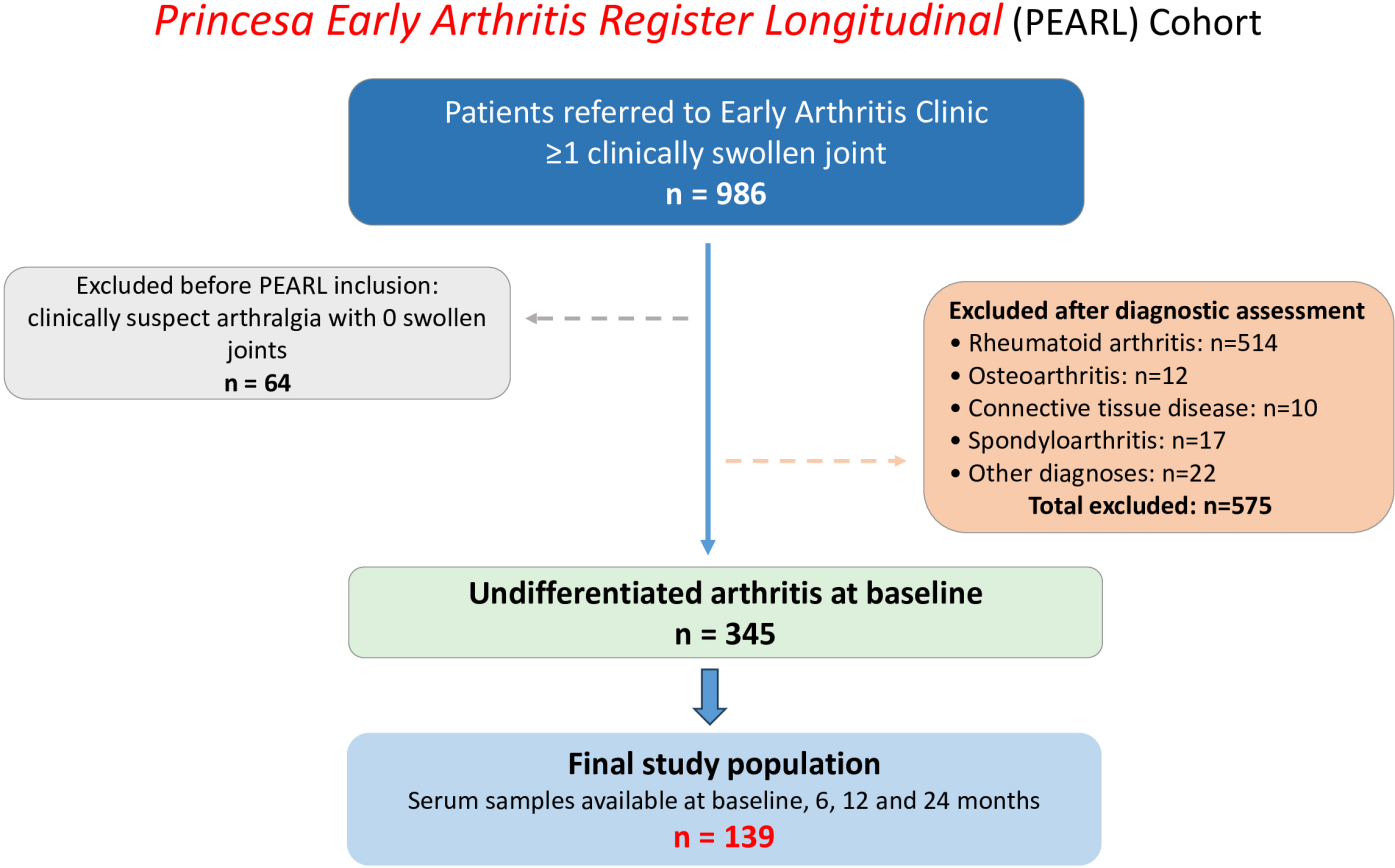
Flow diagram of patient selection from the PEARL cohort. Patients referred to the Early Arthritis Clinic with at least one clinically swollen joint were screened for inclusion in the PEARL study. Patients with clinically suspect arthralgia were excluded prior to registry inclusion. After diagnostic assessment, patients with defined rheumatic diseases were excluded. The final study population included patients classified as undifferentiated arthritis at baseline with available serum samples from all four predefined visits.

For the present study, we selected patients classified as UA at the baseline visit, according to the previously established definition (3), after exclusion of alternative diagnoses including RA, SpA, CTD, crystal-induced arthritis, septic or viral arthritis, and osteoarthritis. For RA classification during follow-up, the PEARL study applied the 1987 ACR criteria, without requiring the 12-week symptom duration component (13). Only patients who completed all four predefined visits (baseline, 6, 12 and 24 months) and had available serum samples from each visit were included to ensure complete longitudinal assessment. A flow diagram summarizing patient selection, exclusions, and final inclusion in the analysis is provided in **Figure 1**.

### 2.2 Measurement of serum Gal-1 levels in UA population

Serum Gal-1 levels were measured longitudinally at all four visits using the Human Galectin-1 Quantikine ELISA Kit (DGAL10; R&D Systems, Minneapolis, USA) following the manufacturer’s instructions. Samples from each patient were tested in duplicate on the same ELISA plate to avoid individual inter-assay variability. In addition, all kind of final diagnosis were included in each ELISA plate in order to minimize bias due to inter-assay variability that was adjusted by including the variable “plate” in the multivariable analysis. After completing the ELISA procedure, plates were immediately read using a spectrophotometer (Innogenetics Diagnóstica y Terapéutica S.A.U., Barcelona, Spain). Absorbance was read at 450 nm with a 620 nm correction.

### 2.3 Variables and statistical analysis

Demographic, clinical, laboratory (including sample collection time), and treatment data were prospectively collected across visits. Disease activity was assessed using DAS-28-CRP score and functional disability with the Health Assessment Questionnaire (HAQ score). As a surrogate variable of disease severity at 24 months of follow-up, we used the Intensity of Disease-Modifying Anti-Rheumatic Drugs (DMARD) Treatment (IDT) score, a weighted treatment intensity score that we have previously described (14). IDT ranged from 0 to 4.76, with median 1,48 and the interquartile range 0 to 1.85. Patients were classified as having mild, moderate or severe disease when IDT score was <=1, >1 and <=2 or >2 points, respectively.

First, baseline Gal-1 serum levels were compared with different outcome variables namely definite diagnosis at 24 months visit, baseline disease activity, use of DMARD treatment and IDT score. In addition, Gal-1 levels across the follow-up were compared with the evolution of disease activity in patients with a final diagnosis of inflammatory rheumatic disease.

Descriptive analyses were performed using appropriate univariate tests. Categorical variables were compared using Fisher’s exact or Chi-square tests, and continuous variables with non-parametric tests (Mann–Whitney U or Kruskal–Wallis). Correlations were assessed with Spearman’s coefficient, and trends across ordered groups with Cuzick’s test. Results were illustrated with boxplots and scatter plots.

For multivariable analyses involving repeated measures, generalized estimating equation (GEE) models (xtgee) were fitted to evaluate longitudinal changes in Gal-1 levels and their association with disease activity, adjusting for potential confounders such as inter-assay variability (plate effects) and duration of frozen storage, previously shown by our group to affect Gal-1 measurements (12). All analyses used Stata 14.0 (StataCorp LP, College Station, TX), with significance set at p<0.05. Given the hypothesis-driven nature of the analyses and the use of unified longitudinal models to assess overall associations across follow-up, formal correction for multiple testing was not applied.

## 3 Results

### 3.1 Patient selection and baseline characteristics

A total of 986 patients from the PEARL study were screened. After excluding alternative diagnoses, 345 patients fulfilled criteria for UA at the baseline visit, of whom 139 were included in the final analysis (**Figure 1**). Overall, the UA cohort was predominantly female (79.8%), with a median age above 50 years, and more than half were ever smokers (**Table 1**). At baseline, 22.3% of patients showed double seropositivity for RF and ACPA and functional disability was generally mild (HAQ = 0.63 [IQR 0.13–1.12]). Disease activity at study entry was moderate to high (median DAS-28-CRP 3.73 [2.44-4.40]).

**Table 1:** Characteristics and treatment requirements of UA patients in the overall cohort and by the type of final diagnosis.

Final diagnosis	Overall N = 139	RA N = 42	Persistent UA N = 56	SpA N = 12	CTD N = 8	Other N = 21	p-value
Baseline characteristics							
Female; n (%) Male; n(%)	111 (79.86) 28 (20.14)	34 (80.95) 8 (19.05)	42 (75) 14 (25)	10 (83.33) 2 (16.67)	7 (87.5) 1 (12.5)	18 (85.71) 3 (14.28)	0.862
Age; p50 [p25-p75]	52.72 [41.70-61.94]	54.69 [50.05– 58.81]	55.21 [42.53 – 63.42]	40.41 [34.02 – 54.81]	45.08 [37.89-56.85]	45.36 [36.21 – 59]	0.034
Obesity (BMI >30); n (%)	21 (15.56)	6 (14.28)	10 (17.86)	2 (16.66)	1 (12.5)	2 (10.53)	0.967
Smoker or ex- smoker	78 (58.65)	26 (66.67)	32 (59.26)	6 (50)	5 (62.20)	9 (45)	0.555
RF positive; n (%) ACPA positive; n (%)	53 (43.80) 39 (32.77)	29 (69.05) 26 (63.41)	23 (41.07) 13 (23.64)	1 (8.33) 0 (0)	2 (25) 0 (0)	0 (0) 0 (0)	<0.001 <0.001
CRP (mg/dl); p50 [p25-p75]	0.3 [0.1-0.73]	0.34 [0.2-0.7]	0.3 [0.11-0.95]	0.55 [0.21-1.08]	0.33 [0.1-0.87]	0.2 [0.1-0.45]	0.361
DAS28; p50 [p25-p75]	3.73 [2.44-4.40]	3.60 [2.24– 4.65]	3.60 [2.35– 4.37]	4.52 [3.68– 5.84]	3.81 [3.04-4.01]	3.73 [2.89–4.05]	0.457
HAQ	0.625 [0.125-1.12]	0.87 [0-1.37]	0.5 [0.18-1]	0.93 [0.18-1.43]	0.87 [0.25-1.25]	0.625 [0.25-0.75]	0.553
Treatment at 2-year follow-up							
Any DMARD;n (%)	101 (72.6)	41 (97.61)	41 (73.21)	10 (83.33)	6 (75)	3 (14.2)	<0.001
MTX; n (%)	77 (55.39)	39 (92.86)	27 (48.21)	7 (63.64)	2 (25)	2 (9.52)	<0.001
LFN; n (%)	18 (12.9)	13 (30.95)	5 (8.93)	0 (0)	0 (0)	0 (0)	<0.001
SSZ; n (%)	12 (8.63)	4 (9.52)	2 (3.57)	4 (36.36)	2 (25)	0 (0)	0.001
HCQ; n (%)	32 (23.02)	10 (23.81)	16 (28.57)	0 (0)	5 (62.5)	1 (4.76)	0.001
Biological therapy; n (%)	4 (2.87)	2 (4.76)	1 (1.78)	1 (8.33)	0 (0)	0 (0)	0.422
Severity							
Mild (IDT<1)	47 (33.81)	2 (4.76)	23 (41.07)	2 (16.67)	4 (50)	16 (76.19)	0.285
Moderate (IDT = 1-2)	64 (46.04)	27 (64.29)	31 (55.36)	4 (33.33)	2 (25)	0(0)	0.485
Severe (IDT>2)	28 (20.14)	13(30.95)	2 (3.57)	6 (50)	2 (25)	5 (23.81)	0.166

Data are presented as n (%) for categorical variables and as median [25th–75th percentile] for continuous variables. Statistical comparisons were conducted using Chi-square, Fisher’s exact, or Kruskal–Wallis tests, as appropriate.

ACPA, Anti-citrullinated protein antibodies; CRP, C-reactive protein; CTD, Connective Tissue Disease; DAS28, Disease Activity Score 28 joints; DMARDs, Disease modifying anti-inflammatory drugs; HAQ, Health Assessment Questionnaire; HCQ, Hydroxychloroquine; IDT, Intensity of DMARD Treatment score; LFN, Leflunomide; MTX, Methotrexate; RA, Rheumatoid arthritis; RF, Rheumatoid factor; SpA, Spondyloarthritis; SSZ, Sulfasalazine; UA, Undifferentiated arthritis.

**Figure 2:**
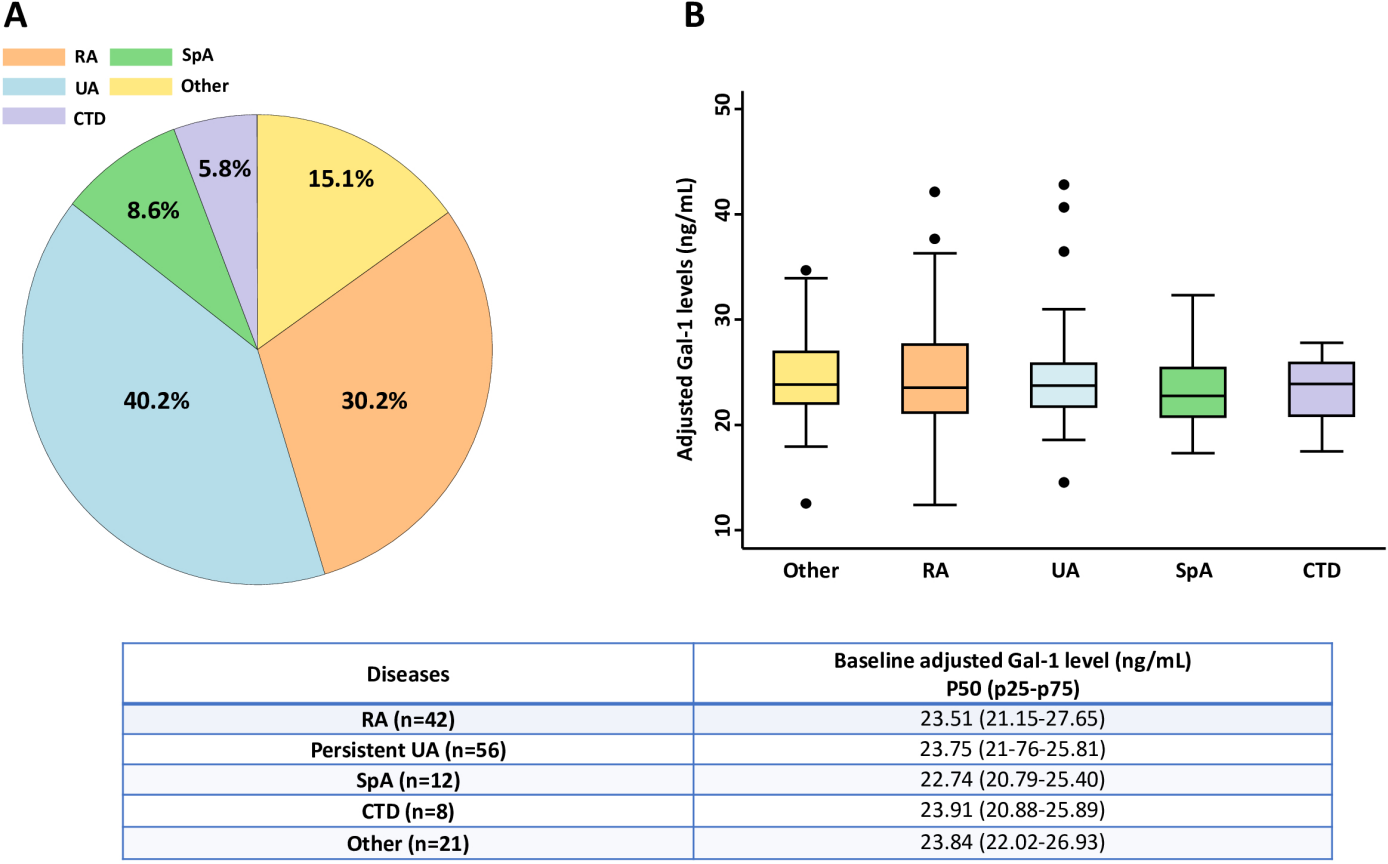
Distribution of baseline serum Gal-1 levels stratified by disease category. **(A)** Proportion of patients by disease category in the study population. **(B)** Baseline plasma Gal-1 levels (adjusted for plate and frozen storage) across disease categories. Boxplots represent the median and interquartile range (25th–75th percentile), with individual patient values overlaid as dots. Group comparisons were performed using the Kruskal–Wallis test. Significance threshold was set at p < 0.05. CTD, connective tissue diseases; Gal-1, galectin-1; RA, rheumatoid arthritis; SpA, spondyloarthritis; UA, undifferentiated arthritis.

After two years of follow-up, 44.6% (n = 62/139) of patients progressed to a definite chronic inflammatory rheumatic disease, most commonly RA (67%; n=42/62), while 40.2% (n = 56/139) remain as UA (**Figure 2A**). The remaining patients (15.1%; n = 21/139) were classified as other conditions, mainly reactive arthritis or osteoarthritis. No significant differences in sex distribution or baseline disease activity parameters were observed across final diagnostic groups. However, patients classified as CTD, SpA or other diagnoses were younger than those with RA or persistent UA.

During follow-up, 72.6% patients received DMARD therapy. Based on the intensity of DMARD treatment score (IDT) score, 20.1% of patients were classified as having severe disease (IDT >2), most frequently among those ultimately diagnosed with SpA (50.0%) or RA (31.0%).

### 3.2 Baseline serum Galectin-1 levels do not correlate with final diagnosis or severity

Baseline serum Gal-1 levels, adjusted for inter-assay variability (plate effects) and duration of frozen storage, showed a modest, non-significant increase with age (approximately 0.2% per year, p = 0.094). No significant differences were observed according to sex, BMI, or other demographic variables. Importantly, adjusted baseline Gal-1 levels were not associated with disease-related characteristics, including seropositivity for RF or ACPA (p = 0.728), CRP levels (p = 0.440), functional disability assessed by HAQ (p = 0.799), or baseline disease activity measured by DAS28-CRP (p = 0.677).

When patients were stratified according to final diagnosis, baseline adjusted Gal-1 levels did not differ significantly across diagnostic groups (p = 0.892), and were comparable between patients who progressed to a defined chronic inflammatory rheumatic disease (RA, SpA, and CTD) and those with persistent UA (p = 0.982) (**Figure 2B**). Although predicted median Gal-1 concentrations tended to be lower in patients evolving to SpA, these differences did not reach statistical significance. Baseline serum Gal-1 levels were also unrelated to disease severity, as assessed by the IDT score. No significant differences were observed between patients with mild versus severe disease (p = 0.872), nor between patients who received DMARD therapy and those who remained untreated during follow-up (p = 0.389).

### 3.3 Galectin-1 serum levels do not correlate with disease activity across the follow-up in patients with confirmed inflammatory rheumatic diseases

**Figure 3:**
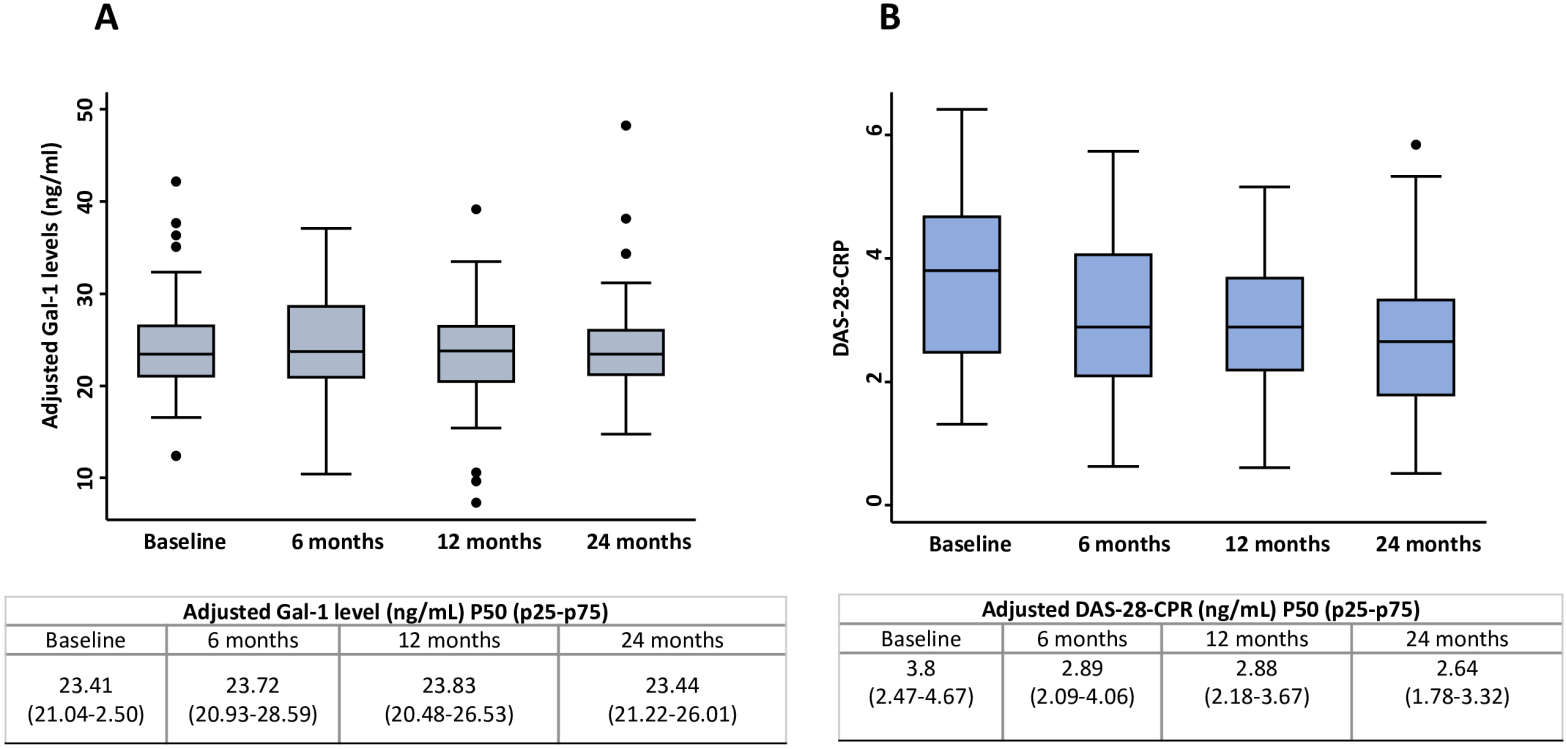
Evolution of adjusted serum Gal-1 levels and DAS-28-CRP scores in patients with chronic inflammatory rheumatic diseases (SpA, RA and CTD) along two years of follow-up. Data are presented as boxplots, showing medians, interquartile ranges (IQR), and whiskers extending to 1.5 × IQR, depicting the evolution of adjusted Gal-1 **(A)** and DAS-28-CRP **(B)** across four visits over a two-year follow-up. Trends in Gal-1 and DAS-28-CRP were assessed using Cuzick’s non-parametric test, with significance defined as p-trend < 0.05. CTD, connective tissue diseases; DAS-28-CRP, Disease Activity Score in 28 joints using by C reactive protein; Gal-1, galectin-1; RA, rheumatoid arthritis; SpA, spondyloarthritis; UA, undifferentiated arthritis.

In longitudinal assessments restricted to patients with defined chronic inflammatory rheumatic diseases (RA, CTD and SpA), adjusted serum Gal-1 levels remained stable throughout the two-year follow-up period (**Figure 3A**; p = 0.770). When analyses were extended to include patients with persistent UA and other final diagnoses (**Supplementary Figure 1**), Gal-1 levels similarly remained stable over follow-up within each diagnostic subgroup, with no evidence of differential longitudinal trajectories across diseases (all group-by-visit interaction p values > 0.05).

**Figure 4:**
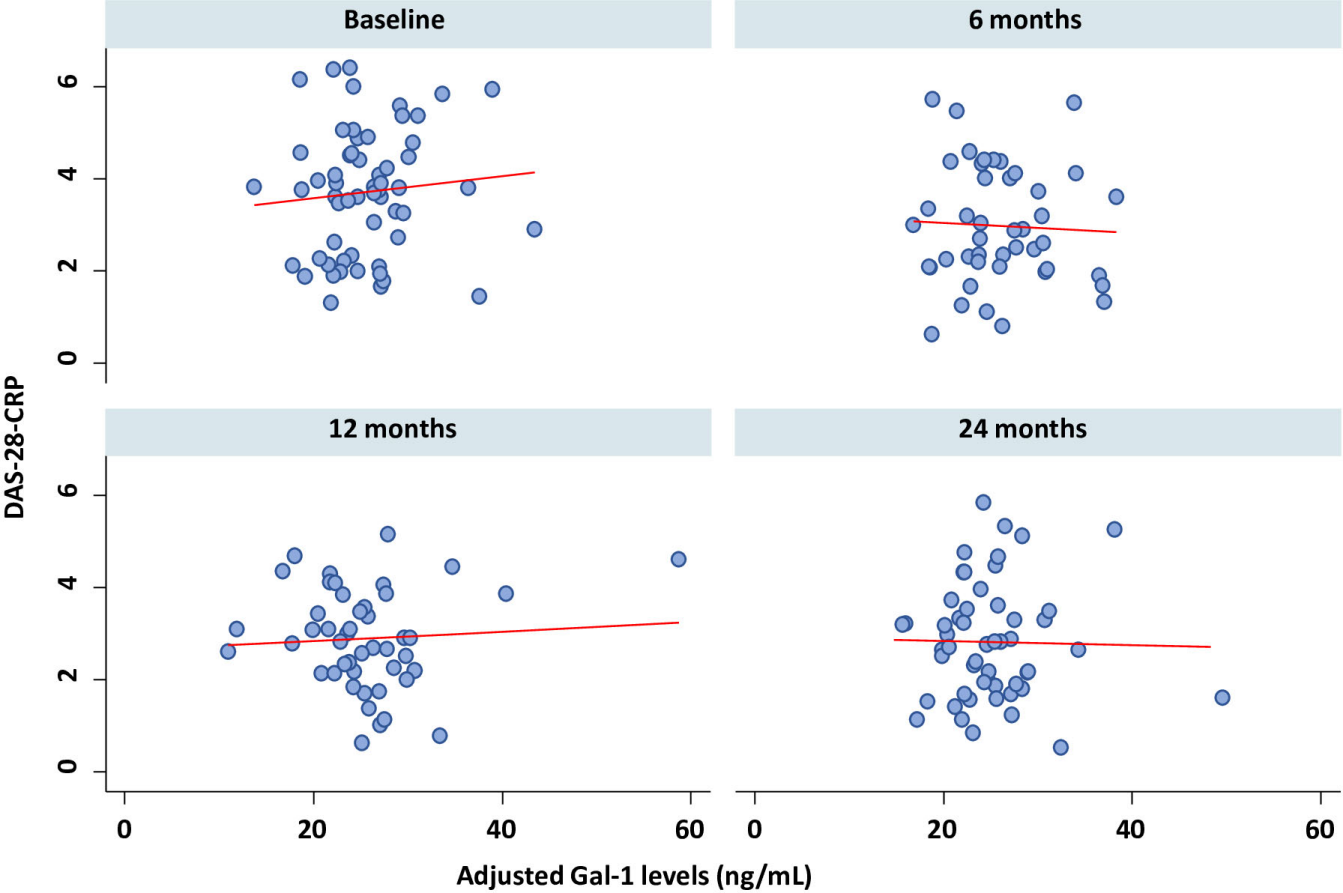
Correlation between Gal-1 levels and disease activity across visits. Scatter plots illustrate the Spearman correlation between Gal-1 and DAS-28-CRP at baseline, 6 months, 12 months, and the final 24-month visit. DAS-28-CRP, Disease Activity Score in 28 joints using by C reactive protein; Gal-1, galectin-1.

In contrast, disease activity showed a significant improvement over time, as reflected by a progressive decrease in DAS-28-CRP scores across follow-up visits in the overall inflammatory cohort (**Figure 3B**; **Supplementary Table 1**; p < 0.001) and within individual diagnostic groups (all p < 0.05, except CTD: p = 0.074). Accordingly, no significant correlation was observed between serum Gal-1 levels and DAS-28-CRP across follow-up visits, either in the overall cohort or within diagnostic subgroups (**Figure 4**; all p > 0.30 and **Supplementary Figures 1**, **2**).

## 4 Discussion

This study provides the first longitudinal evaluation of serum Gal-1 levels in patients initially diagnosed with UA over a two-year follow-up. Our results indicate that baseline Gal-1 serum levels did not predict progression from UA to a definite diagnosis (RA, SpA, or CTD), arthritis severity, or treatment requirements. Longitudinal analyses also did not support Gal-1 as a biomarker of disease activity.

Previous studies have highlighted the immunomodulatory and anti-inflammatory properties of Gal-1 in autoimmune diseases, suggesting its potential as a biomarker in RA or SLE (11). While most studies reported elevated serum Gal-1 levels compared to healthy controls in both early (12) and established RA (15–17), some found no significant differences (18–20). In SLE, our recent study demonstrated elevated Gal-1 levels compared with controls (18), whereas no differences were observed in psoriatic arthritis (PsA), consistent with the findings of Bably et al. (21). To our knowledge, no previous studies have assessed Gal-1 modulation across different follow-up stages in an overall UA cohort.

In our cohort, UA patients who subsequently developed a chronic inflammatory rheumatic disease (RA, CTD, or SpA) within two years exhibited similar Gal-1 levels at baseline and throughout follow-up, with no significant variation across diagnostic outcomes. No differences were observed between RA and persistent UA, in line with our previous study in the overall early arthritis population (12), although a tendency toward lower levels was noted in UA, which represented only 34% of the sample. These findings may reflect the very early stage of disease in this population, where inflammatory mechanisms are less active and Gal-1 levels remain relatively low. Supporting this notion, we recently reported that Gal-1 concentrations are influenced by disease duration and may be associated with disease severity in long-term RA patients (18). In that study, patients with immune-mediated inflammatory diseases (IMIDs), including RA, SLE, and PsA with longer disease duration (>5 years) showed significantly higher plasma Gal-1 levels than patients with shorter disease duration.

Nonetheless, UA patients who later evolved to SpA presented slightly lower Gal-1 levels compared to other diseases, although differences were not statistically significant. This observation may reflect distinct immunopathological pathways and differential regulation of Gal-1 in SpA compared with RA or CTD. Consistently, previous studies have reported higher Gal-1 levels in RA than in SpA or PsA, both in serum and synovial fluid (18, 21, 22). The divergent immunoregulatory roles of galectins across IMIDs may underlie these findings. Gal-1 primarily exerts anti-inflammatory effects by inhibiting Th1 and Th17 responses and promoting Treg expansion, but it can also favor Th2 polarization and humoral activation, enhancing B-cell differentiation and antibody production (8–10). This dual behavior may contribute to elevated levels in diseases dominated by adaptive and humoral immune activation, such as established RA or SLE, where Gal-1 may act as a compensatory regulatory molecule. Conversely, the relatively low Gal-1 levels observed in SpA could reflect the predominance of innate and IL-23/IL-17–driven immune pathways (23), in which Gal-1 expression and signaling appear less central (8–10).

In this study, baseline serum Gal-1 levels were not significantly associated with other disease parameters or severity indicators, including seropositivity or the need for intensive treatment. Similarly, other studies have failed to demonstrate a link between Gal-1 levels, radiographic progression, seropositivity, or response to DMARD therapy (16, 19, 22). Gal-1 levels were independent of disease activity over the two-year follow-up, despite improvements in DAS-28 scores, agreeing with previous reports in early and established RA (12, 16, 17, 19, 21) and early SpA (22). In contrast, Méndez-Huergo et al. (15) reported a positive correlation in two independent cohorts of 80 RA patients with long-standing, high-activity disease, consistent with our recent study in IMIDs (18), in which only late-stage patients showed an association between Gal-1 levels and disease activity.

Our study has several limitations. First, this was a single-center study, limiting the generalizability of the results, as the cohort was predominantly white European and patient management may have been influenced by local clinical practices. Second, the limited sample size and uneven distribution of patients across final diagnostic groups may have reduced statistical power. Third, the follow-up period was limited to 24 months, which although generally appropriate for early diagnosis in RA, axSpa or PsA (24–26), may be insufficient to fully capture delayed diagnostic transitions in other inflammatory rheumatic diseases such as peripheral SpA (27) or CTD (28), where prodromal or clinically subtle phases may be prolonged beyond two years. Finally, patient samples were collected many years ago (some over 20 years), which may have impacted Gal-1 detection, as prolonged frozen storage can significantly reduce measurable levels, as previously reported by our group (12). We attempted to mitigate this effect by adjusting for storage duration in our analyses.

Future studies should be conducted in larger, prospective, multicenter cohorts using standardized ELISA techniques to validate these findings. Additionally, the observed trend toward lower Gal-1 levels in SpA patients warrants further investigation in larger samples, as the current cohort may not fully capture potential associations or predictive patterns.

## 5 Conclusion

In conclusion, although Gal-1 is involved in immune-regulatory pathways relevant to RA, SpA, and SLE pathogenesis, our findings indicate that it has limited value in predicting which UA patients will progress to established chronic inflammatory rheumatic disease. Baseline and longitudinal Gal-1 levels did not correlate with disease activity, prognosis, or diagnostic outcomes. Notably, a trend toward lower Gal-1 levels was observed in patients evolving to SpA, although the small sample size limits interpretation. These findings suggest variable regulation of Gal-1 across distinct pathogenetic mechanisms rather than reflecting overall inflammatory burden.

## Data Availability

The raw data supporting the conclusions of this article will be made available by the authors, without undue reservation.
