# Supplementary material for: Galectin-1 levels do not predict outcomes in undifferentiated arthritis: a two-year prospective observational study
Source: Front Immunol. 2026 Feb 11;17:1757168. doi: 10.3389/fimmu.2026.1757168 (PMC12932545; doi:10.3389/fimmu.2026.1757168)
Supplement: Supplementary file 1 [file Table1.docx]

**Supplementary Material.**

**Supplementary Table 1.** Plasma levels of Galectin-1 and DAS-28 score measured at baseline, 6, 12, and 2 years across different disease groups.

| Diseases | RA (n=43) | | Persistent UA (n=56) | | SpA (n=13) | | CTD (n=8) | | Other  (N=19) | |
| --- | --- | --- | --- | --- | --- | --- | --- | --- | --- | --- |
| Time-points **P50  (p25-p75)** | DAS-28-CRP | Gal-1 (ng/mL) | DAS-28-CRP | Gal-1 (ng/mL) | DAS-28-CRP | Gal-1 (ng/mL) | DAS-28-CRP | Gal-1 (ng/mL) | DAS-28-CRP | Gal-1 (ng/mL) |
| Baseline level | 3.6  (2.24-4.65) | 23.51  (21.15-27.65) | 3.6  (2.35-4.37) | 23.75  (21.76-25.81) | 4.52  (3.68-5.84) | 22.74  (20.79-25.40) | 3.81  (3.04-4.01) | 23.91  (20.88-25.89) | 3.73 (2.89-4.05) | 23.84  (22.02-26.93) |
| 6 months | 2.79  (2.14-4.06) | 24.87  (20.14-28.37) | 2.41  (1.7-3.35) | 23.78  (21.41-27.12) | 3.19  (2.08-4.33) | 22.74  (21.21-24.65) | 2.17  (1.67-3.03) | 22.68  (20.64-31.82) | 2.95  (2.2-3.4) | 21.29  (18.61-25.63) |
| 12 months | 2.88  (2.16-3.85) | 24.13  (21.53-27.21) | 2.42  (1.8-3.57) | 23.52  (21-26.05) | 2.67  (1.12-3.83) | 25.06  (16.7-26.19) | 2.92  (1.69-3.68) | 18.65  (16.48-20.48) | 2.48  (1.79-2.95) | 23.64  (20.83-26.4) |
| 24 months | 2.81  (1.9-3.48) | 23.4  (21.1-25.95) | 2.23  (1.37-3.07) | 24.6  (21.65-26.14) | 2.64  (2.16-3.72) | 27.05  (19.59-21.16) | 1.56  (1.12-3.52) | 22.62  (21.88-25.60) | 1.9  (1.67-3.32) | 22.13  (20.09-33.23) |

***Abbreviations:** CTD: connective tissue disease; Gal1: galectin-1; RA: Rheumatoid arthritis; RF: Rheumatoid factor; SpA: Spondyloarthritis; UA: Undifferentiated arthritis.

**Supplementary Figure 1.** Longitudinal evolution of serum galectin-1 levels and disease activity across diagnostic groups.

Panels show the median serum galectin-1 levels (blue line with interquartile range) and median DAS28-CRP scores (red dashed line) across follow-up visits (baseline, 6, 12, and 24 months). ***Abbreviations:** CTD: connective tissue disease; Gal1: galectin-1; IQR: interquartile range; RA: Rheumatoid arthritis; RF: Rheumatoid factor; SpA: Spondyloarthritis; UA: Undifferentiated arthritis.

**Supplementary Figure 2.** Correlation between serum galectin-1 levels and DAS-28-CRP values across follow-up visits.

Scatter plots illustrate the correlation between adjusted serum galectin-1 concentrations and DAS28-CRP scores at baseline, 6, 12, and 24 months. Each panel corresponds to a study visit, and colors indicate final diagnostic groups. ***Abbreviations:** CTD: connective tissue disease; RA: Rheumatoid arthritis; RF: Rheumatoid factor; SpA: Spondyloarthritis; UA: Undifferentiated arthritis.
